# The National Dispensatory

**Published:** 1884-11

**Authors:** 


THE NATIONAL DISPENSATORY. *Containing the Natural History, Chemistry, Pharmacy, Actions and Uses of Medicines; including those recognized in the Pharmacopœias of the United States, Great Britain, and Germany, with Numerous References to the French Codex. By* ALFRED STILLE, M.D., L.L. D., *Professor Emeritus of the Theory and Practice of Medicine and of Clinical Medicine in the University of Pennsylvania, and* JOHN M. MAISCH, PHAR. D., *Professor of Materia Medica and Botany in the Philadelphia College of Pharmacy. Third Edition, thoroughly revised, with numerous Additions. Three Hundred and Eleven Illustrations. Octavo, pp. xiv., 1755. Philadelphia:* HENRY C. LEA'S SON & CO. *1884. Chicago:* JANSEN, MCCLURG & CO. *1884.*

It was predicted in 1879, from *apriori* considerations, that the National Dispensatory would achieve brilliant success. With Professors Stille and Maisch—leaders in their respective professions of Medicine and Pharmacy, as authors, failure was difficult of conception. Two large editions have been rapidly exhausted in the brief period of five years! The authors and publishers could not have anticipated this cordial recognition of a practical book by practical men. The reviewer's task is a supererogation. The third edition of the National Dispensatory embodies the recent editions of the Pharmacopœias of four chief civilized Nations, the United States, the United Kingdom, Germany and France. The illustrations of the present edition—increased in number—are good, while the paper, type, binding, and general appearance of the volume reflect credit upon the intelligence, liberality, and business enterprise of the publishers. W. W. J.

